# Can Fire and Rescue Services and the National Health Service work together to improve the safety and wellbeing of vulnerable older people? Design of a proof of concept study

**DOI:** 10.1186/1472-6963-10-327

**Published:** 2010-12-03

**Authors:** Karen Lowton, Anne H Laybourne, David G Whiting, Finbarr C Martin

**Affiliations:** 1 King's College London, Institute of Gerontology, Strand, London, UK; 2 London Fire Brigade, Union Street, London, UK; 3 Department of Ageing and Health, Guy's and St Thomas' NHS Foundation Trust, London, UK

## Abstract

**Background:**

Older adults are at increased risk both of falling and of experiencing accidental domestic fire. In addition to advanced age, these adverse events share the risk factors of balance or mobility problems, cognitive impairment and socioeconomic deprivation. For both events, the consequences include significant injury and death, and considerable socioeconomic costs for the individual and informal carers, as well as for emergency services, health and social care agencies.

Secondary prevention services for older people who have fallen or who are identifiable as being at high risk of falling include NHS Falls clinics, where a multidisciplinary team offers an individualised multifactorial targeted intervention including strength and balance exercise programmes, medication changes and home hazard modification. A similar preventative approach is employed by most Fire and Rescue Services who conduct Home Fire Safety Visits to assess and, if necessary, remedy domestic fire risk, fit free smoke alarms with instruction for use and maintenance, and plan an escape route. We propose that the similarity of population at risk, location, specific risk factors and the commonality of preventative approaches employed could offer net gains in terms of feasibility, effectiveness and acceptability if activities within these two preventative approaches were to be combined.

**Methods/Design:**

This prospective proof of concept study, currently being conducted in two London boroughs, (Southwark and Lambeth) aims to reduce the incidence of both fires and falls in community-dwelling older adults. It comprises two concurrent 12-month interventions: the integration of 1) fall risk assessments into the Brigade's Home Fire Safety Visit and 2) fire risk assessments into Falls services by inviting older clinic attendees to book a Visit. Our primary objective is to examine the feasibility and effectiveness of these interventions. Furthermore, we are evaluating their acceptability and value to key stakeholders and services users.

**Discussion:**

If our approach proves feasible and the risk assessment is both effective and acceptable, we envisage advocating a partnership model of working more broadly to fire and rescue services and health services in Britain, such that effective integration of preventative services for older people becomes routine for an ageing population.

## Background

### Risks and consequences of falls and fires

There are around 61 million people resident in the UK, 1 in 6 of whom are aged 65 years or older [1]; in London alone there are nearly 1.2 million older adults. Older people are at an increased risk of both experiencing an accidental domestic fire [2] and falling [3]. Described as one of the 'giants' of geriatric medicine, around one in three older people will fall annually, with half of these falls being recurrent [4]. This represents over 15,000 people a year experiencing a fall in the London boroughs of Lambeth and Southwark alone. Accidental domestic fires also present a considerable danger to older adults, with the highest percentage of fatalities from fire occurring in the over 60 year age group [5], predominantly living in deprived areas. Currently, around 2,700 fires are reported annually in Southwark and Lambeth, with 635 being accidental domestic fires (London Fire Brigade figures for 2006/7).

Factors increasing an older person's risk of falling include: advanced age; reduced lower limb strength; balance deficits; history of falls; multiple and specific culprit medications, particularly sedatives; visual impairment; and cognitive impairment [3,6]. Similarly, reasons for the disproportionately high number of injuries and fatalities from fire in older age groups include physical and cognitive disabilities such as mobility problems; frailty; dementia; and medication use. Additionally, the possibility of unintentionally setting light to clothing in an intended residential fire [7] ensures that fire safety is currently an important albeit often unacknowledged part of the informal carer role, for example ensuring electric fires are turned off, or burning cigarettes are disposed of safely. Living arrangements also influence domestic fire risk, with old housing [8] and single person households identified as risk factors. Three in five women aged 75 years or older live alone, [9] putting them at particular risk of harm from accidental domestic fires. Furthermore, a social class gradient to fire injury exists in the older population with people in lower income brackets at increased risk [10].

The consequences of fires and falls for older people are considerable and, as with the risk factors, are in many ways similar. Falling can result in a range of injuries including fractures and death; 62% of all fatal injuries in people aged 65 years and older are the result of a fall [11]. Furthermore, there may be psychological and social consequences including increased fall-related fear or reduced confidence, reduced social participation or network size. Both falls and fires have important and considerable socioeconomic costs for the older population, including personal injury; loss of income to informal carers; health and social care costs; and property damage or loss. The financial cost of falling is also substantial, estimated at nearly £1 billion per annum. This represents costs to Personal Social Services of £400 million and £581 million to the NHS. The majority of these costs are attributable to falls in adults aged 75 years and older [11]. Put in context, the total cost of falling described here is the equivalent of almost 20% of total NHS pharmaceutical expenditure, the total budget of a new strategic health authority, or more than three times the earmarked budget for mental health, coronary heart disease, cancer, and primary care in England [11].

Fire also imposes a significant cost on the UK economy. In 2004, the total cost in terms of anticipation, response and consequences of fire was estimated at £7.03bn for England and Wales, equivalent to approximately 0.78% of the gross value added of the economy, a measure of total national output [12]. The average cost of an accidental domestic fire is estimated at £24,900, of which approximately £14,600 is accounted for by the economic cost of injuries and fatalities, and £7,300 due to property damage.

It is clear that early prevention of both falls and accidental domestic fires amongst community-dwelling older people could have significant and positive financial implications; the Mayor of London's Older People Strategy [13] aims to improve fire safety in the home and the health and social care of older adults in London. Specifically, the National Service Framework for Older People [14] aimed to reduce the number of falls and fall-related injuries. In response, falls prevention is now an important strand of clinical gerontological research and as a result, interventions such as evidence-based exercise programmes are the mainstay of NHS falls prevention and rehabilitation strategies [15]. Rate of falling is reduced by 20% [16] following such interventions. Other outcomes may include improved balance and gait [17] and reduced fear of falling, although evidence here is inconsistent [18]. Guidance from the National Institute of Health and Clinical Excellence (NICE) recommends that health professionals should routinely ask older people about whether or not they have fallen in the previous year and enquire as to the nature and frequency of falls [19], and that older people admitted to hospital following a fall should be offered a falls home hazard assessment, with safety advice and environmental modification as appropriate.

In a similar vein, successful working over the past three decades to reduce mortality from home fires has led British Fire and Rescue services to carry out community safety interventions to reduce injury from fires, for which they now have a statutory duty under the Fire and Rescue Services Act (2004). Duties under the Act include the promotion of fire safety in the area, including the provision of advice in respect to the prevention of fire and escape from a fire, should one occur. In response to the Mayor of London's Older People Strategy the London Fire Brigade (LFB) has developed its own Older People Strategy [2]. The Strategy specifically aims to improve the fire safety of older people with the explicit commitment to continue and expand older adults' community safety initiatives, including provision of Home Fire Safety Visits. These Visits target vulnerable people living throughout London, where fires and fire deaths are high. LFB, for example, concentrates on a 'places and faces' approach to risk reduction in the community by focusing on where and who are most at risk in terms of likelihood and consequences of fire. The intervention includes a discussion of fire safety in the home, fitting of a free smoke alarm, instruction regarding regular testing and maintenance of the alarm, and planning an escape route in the event of accidental fire. Recent evaluation of similar schemes in North America suggest that fitted alarms account for up to 80% of the reduction in accidental fire injury [20].

### Bringing falls and accidental domestic fire prevention together

Typically, partnerships between the Fire and Rescue and health services focus on staff responding to community-based emergencies rather than prevention strategies; for example North American fire fighters are qualified to at least Basic Emergency Medical Technician level, and in North America and Australia, fire fighters trained as 'first responders' use automatic external defibrillators at medical emergencies occurring in the community [21,22]. In the UK, emergency medical training may occur alongside fire fighting duties in some Fire and Rescue services [23], yet no national standard currently exists for the level of fire fighters' medical knowledge and skills [23], nor understanding of how these might be used more generally in a preventative community-based approach.

Somewhat surprisingly, given the shared population, risk factors, consequences of falling and accidental domestic fire, and services' emphasis on preventative work, preventative joint working between the Fire and Rescue services and NHS has not, to our knowledge, been investigated in the UK. This is despite the Fire and Rescue Service National Framework 2008 - 2011 [24] encouraging Fire and Rescue Authorities to work locally with partners to identify targets that are priorities within their local area and to offer appropriate contributions, both in terms of time and resources, to meet these. Within the Framework a strong emphasis is placed on building community safety to prevent emergencies occurring, and on working with other providers to improve 'life safety' services. With encouragement from the UK government for partnership working between local public services [25]; its commitment to a greater emphasis on prevention of avoidable morbidity and disability [26] and fire fighters positively evaluating an increased skill base [22], substantial personal, social and economic benefits of joint working in the community might now be achievable.

### Objective

We have designed an intervention to promote closer working in preventative care between the Fire and Rescue Service and the NHS. This is a proof of concept study to evaluate the potential of implementing two concurrent interventions with the purpose of further reducing the risk of accidental domestic fires and of falling amongst older adults living in Lambeth and Southwark.

The Home Fire Safety Visit serves as an important new referral mechanism for older adults currently unknown to Falls services but likely to be at high risk of hospitalisation or injury due to a fall. Given its focus on vulnerable community-dwelling older people, the Visit scheme offers a potential opportunity for joint working through the inclusion of a falls risk assessment. Equally, Falls clinics attendees are potentially a vulnerable group with high domestic fire risk; attendance at the clinic offers the opportunity to deliver home fire safety information and promote the benefit of a Home Fire Safety Visit.

The specific study objectives are:

1. Can the Home Fire Safety Visit be used effectively to assess older people's functional and environmental risks of falling?

2. Can the Home Fire Safety Visit be used to identify and refer appropriately older people to a specialist Falls service?

3. Is the Falls service an effective context for the referral of older people to LFB?

4. How acceptable is this joint working to (i) service users (ii) LFB staff, (iii) NHS Falls service staff?

## Methods/Design

### Settings

The research is located in Southwark and Lambeth, ranked respectively as the 6^th ^and 8^th ^most deprived London boroughs. These boroughs have a culturally diverse profile, with around a third of the population in Southwark from minority ethnic groups, and between 11-13% of the population aged 60 years and over (Table 1[27,28]). Lambeth across all the London boroughs has the highest average annual cost of fires in private domestic dwellings (£10.2 million), with costs in Southwark, ranked 11^th^, estimated at £7.2 million per annum [29].

**Table 1:** Southwark and Lambeth Borough Profiles

	Southwark	Lambeth
Area (km^2^)	28.9	26.8
Total population	257 675	269 127
Number of people per km^2^	8932	10 035
Density ranking in London	9/33	8/33
Population aged 60 and over	32 964 (13%)	31 016 (11%)
Black and ethnic minority population	37%	38%
Deprivation ranking in London (1 = most deprived)	6/33	8/33
Overcrowding ranking in London (1 = most deprived)	8/33	13/33
Public sector owned housing	53%	41%
Emergency fire calls ranking in London	4/33	17/33
Accidental fires in dwellings 2006/7	307	328
Injuries arising from accidental fires in dwellings 2006/7	53	47
Home Fire Safety Visits completed 2006/7	1059	1428
Smoke alarms fitted 2006/7	1911	1961

Profiles of Southwark and Lambeth boroughs, taken from London Fire Brigade's Borough Profiles 2007 [27,28]

We have chosen Southwark and Lambeth as two boroughs with particularly vulnerable populations; although we acknowledge the problems of reaching vulnerable or excluded groups, we are confident that we will be able to recruit a significant number to our study. There are four NHS Falls clinics and nine fire stations (one being a fire boat station) within these boroughs.

#### London Fire Brigade

Southwark borough has four fire stations (Dockhead, Old Kent Road, Peckham, and Southwark), as does Lambeth (Brixton, Clapham, Lambeth, and West Norwood). Each station, crewed around the clock by four watches (shifts) of fire fighters, has an annual target to carry out approximately 400 Home Fire Safety Visits; Lambeth and Southwark therefore have around 3200 Visits to complete per annum. The fire brigade in each Borough take a different approach to achieving their Home Fire Safety Visit targets; Lambeth uses a referral method while Southwark uses a targeted method. Lambeth stations identify available 90 minute Visit slots up to two months in advance which are used by the central call centre at Brigade Headquarters to schedule residents' requests for a Visit. Cancelled Visits are also logged here for one of five reasons: (i) fire engine mobilised to an emergency incident; (ii) fire engine ordered to provide fire cover at another fire station, therefore away from its usual station ground; (iii) due to a large number of incidents or a major incident, outside activities such as Home Fire Safety Visits can be cancelled to allow staff to focus on emergency responses; (iv) fire engine defective or unable to respond safely (v) resident unavailable.

The Brigade and the fire brigade Borough Commanders work with pan-London and local agencies to identify and access individual older people who may be vulnerable. This has previously been achieved via arrangements with, for example, Age Concern, Help the Aged (now joined together as Age UK), housing associations, primary medical care centres, Local Authority departments, mobility scooter shops, and bingo halls. Other social marketing techniques such as advertising aimed at older people on television and radio has also proved effective, as has advertising the Home Fire Safety Visit service on pharmacy bags. Additionally, the Visit scheme is advertised on the Brigade's fire engines, website and in parts of the community where the most vulnerable people or their carers may visit, for example doctors' surgeries, chemists, and libraries.

Stations within Southwark carry out their Home Fire Safety Visits in a targeted fashion; addresses in the borough most at risk of experiencing accidental domestic fires are identified using a number of datasets including historic fire data and predictive tools such as MOSAIC, a classification of residential postcodes in the UK which is essentially a "geodemographic" tool. MOSAIC uses a combination of census, electoral roll, housing and financial data (86 variables in all) to classify households into 12 lifestyle groups and 52 sub-groups. This allows targeting to take place at a street or postcode level. Each quarter, fire fighters from each Southwark station identify time slots in which to target an area and essentially 'door knock', offering an immediate Home Fire Safety Visit to those residents present on the day. Crews will endeavour to return to the same area until all residents have been approached.

#### Falls clinics

One of our interventions will allow targeting of another group which may be deemed vulnerable; those who have already experienced a fall or mobility problems. Four Falls clinics exist within the borders of Southwark and Lambeth: King's College Hospital, Dulwich (south Southwark, run by Kings College Hospitals trust); Whittington Centre, Streatham (south Lambeth, run by Lambeth primary care trust); Lambeth Community Care Centre, Kennington (north Lambeth, run by Lambeth primary care trust); and Guy's Older Peoples' Assessment Unit at Guy's Hospital, London Bridge (north Southwark, run by Guy's and St. Thomas' NHS Foundation Trust). While managed separately, these clinics form part of a local NHS and local authority integrated pathway for the prevention of falls (*SLIPs *= **S**outhwark and **L**ambeth **I**ntegrated Care **P**athway for Falls **P**revention, http://www.slips-online.co.uk).

Patients who have fallen or who are deemed at risk of falling are referred to these clinics via a number of routes, most commonly from hospital Accident and Emergency departments, general practitioners, or community social service departments, using consistent referral criteria and assessment approaches as part of the integrated pathway. These referral criteria are:

• People who present to hospital following a fall, whether or not injury is present.

• Those identified by clinicians as recurrent fallers

• Those identified by clinicians as having mobility problems and likely to fall

At clinic, an individual undergoes a structured multi-factorial risk assessment, with the multidisciplinary clinical team working with the patient to agree a suitable set of actions. Outcomes for the patient following clinical assessment commonly include a domiciliary or group based falls prevention strength and balance exercise programme, along with one or more of several targeted approaches such as (i) home safety modifications (ii) medication modification, (iii) referral for management of vision problems.

#### Study participants

We are recruiting people aged 60 years and over who are living in private or sheltered housing in Southwark and Lambeth via two routes: 1) those in contact with LFB for the purposes of receiving a Home Fire Safety Visit, including those referred by an external agency and 2) those attending any of the four Falls clinics who have not had a Home Fire Safety Visit in the past year.

#### Recruitment through Home Fire Safety Visits

In the Home Fire Safety Visit arm of the study, we are recruiting older people living in the community who have either pre-booked a Visit from LFB or are approached during targeted calling. Older people who have pre-booked their Visit receive an information sheet and leaflet sent by the call centre, outlining the project and inviting them to have a falls assessment at or immediately after their booked Home Fire Safety Visit. During targeted calling exercises, residents aged over 60 years old who consent have their details passed to a researcher (AL) are approached with further information about the study and invited to make an appointment for a falls risk assessment. We are recruiting over a 12 month period and anticipate a sample of around 400 participants.

#### Recruitment through Falls clinics

All eligible older people attending an appointment at one of the Falls clinics receive an information leaflet outlining the study. They are also given a booklet [30] containing information of fire risk reduction and the Home Fire Safety Visit. Patients are invited to complete and return a uniquely coded Freepost reply card in their own time, indicating their interest in a Home Fire Safety Visit and thus giving their consent to project participation.

### Interventions and measures

**Figure 1:**
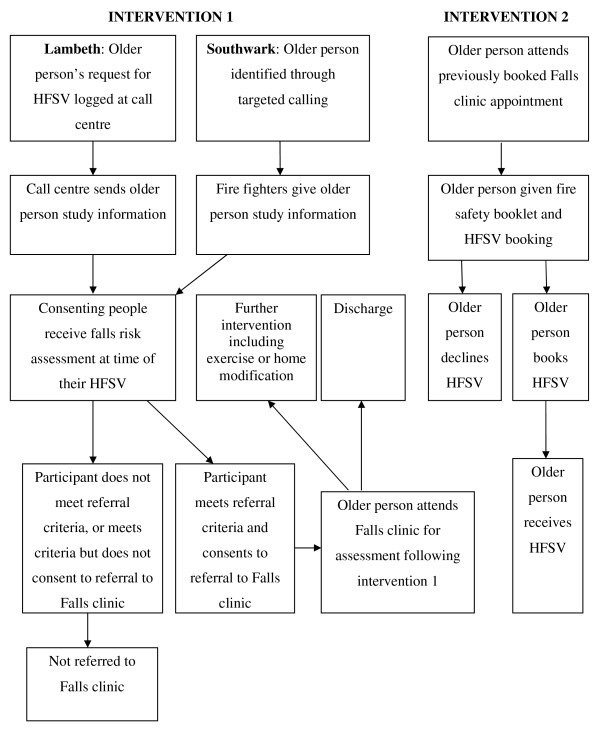
**Recruitment flow chart for Home Fire Safety Visits (HFSV, Intervention 1) and Falls Clinics (Intervention 2)**.

Two concurrent 12 month interventions are being carried out (Figure 1).

Due to seasonal variability in Home Fire Safety Visits, we will count the number of Visits conducted for people aged 60 years and older in Southwark and Lambeth during the year immediately prior to the intervention, to allow comparison and therefore evaluation of the intervention's impact. This will be done through records available at LFB Headquarters. Similarly, we will assess the number and outcome of referrals to Falls clinics in the period prior to the commencement of the Falls clinic intervention. Due to the rate of Falls clinic referrals being relatively stable, we will collect baseline data covering a three month period.

#### Home Fire Safety Visit intervention

An in-depth falls risk, fear, and functional ability assessment is carried out by the researcher in the respondent's home. This comprises a Timed-Up-and-Go (TUG) test, assessing balance and mobility [31]; assessment of fear of falling using the Short Falls Efficacy Scale [32]; and the Home Falls and Accidents Screening Tool (HOMEFAST), a broad home falls and accidents screening assessment to assess falls hazards in the home environment [33,34]. The booklet 'Staying Steady' [35], produced by Help The Aged and endorsed by the British Geriatrics Society, is left with all participants. The booklet contains advice for older people on improving strength and balance as well as information on eyesight, medications, podiatry, and community alarms. Useful contact details are also provided within the booklet.

The falls risk assessment takes less than 20 minutes and is carried out within or immediately following the participant's Home Fire Safety Visit, as appropriate. Based on the result of the falls risk assessment, consenting eligible participants are referred to their closest Falls clinic, based on the postcode system used currently by clinics. The researcher informs the relevant clinic of the participant's contact details and falls and home assessment results. This is then managed in the same way as referrals through more established routes. Participants are eligible for the patient assisted transport service.

#### Falls clinics intervention

All patients aged 60 years and older attending the four Falls clinics receive from their clinic nurse the 'Fire Safety in the Home' booklet [30] which contains information on fire risk reduction and the Home Fire Safety Visit scheme. Clinic staff have been trained to use a standardised message about the booklet and importance of the Visit, to give participants an information leaflet about the intervention, and to mark on the daily clinic list all patients who have received this information. Patients are also given a Home Fire Safety Visit freepost card, to which they (or their carer) are asked to respond by booking a Visit by the Fire Brigade. The service is free to access and receive. An additional Freepost card is appended to the clinic letter posted to every new patient attendee after their appointment.

There are a number of methods for patients to book a Visit besides posting the card: a dedicated email address; freephone and fax numbers; and a minicom number, which is a telephone typewriter device for communication between deaf, hard of hearing, speech-impaired and/or hearing persons. Using a unique project code, LFB control centre log each patient request for a Visit and subsequent appointment that is made through the Falls clinic route. We are then able to track how many bookings result in Visits.

### Key outcome measurements

Measuring outcomes is as important as measuring the process of partnership working [36]. Ultimately this proof of concept study aims to assess whether those at risk of falling and accidental domestic fires can be identified and cross-referred appropriately and efficiently through partnership working, and what factors or systems facilitate or impede this. We will use both quantitative and qualitative methods to evaluate the interventions in terms of process and outcome. Key outcome measures for each of the four study objectives above are:

#### Objective 1

(i) The number of older people identified and assessed though Home Fire Safety Visits who meet the criteria for the Falls services, previously unknown to the Falls services

(ii) The number of older people who have fallen in the previous 12 months

(iii) The number of older people assessed and identified through Home Fire Safety Visits who meet the criteria for the Falls service, previously known to the Falls services

Specifically, we are collecting demographic information about all older people receiving a Home Fire Safety Visit and a study invitation letter; all who refused a combined fires and falls assessment; all who consented but subsequently cancelled a Visit, and the numbers of Visits cancelled and rebooked by LFB. Details of all study participants for whom a falls assessment could not be carried out are noted, with the reasons for failure. We record demographic and assessment data for all older people who meet the criteria for referral to the Falls services, by noting which specific established clinical criteria used by the Falls services they meet.

#### Objective 2

(i) The number of people referred from Home Fire Safety Visit to the Falls services

(ii) The number of people who receive and attend an appointment

(iii) The number of people who receive and fail to attend an appointment

(iv) Falls service outcome e.g. onward referral to physiotherapy, exercise programme

We are collecting all participants' fires and falls assessment data. The Home Fire Safety Visit assessment forms collect socioeconomic data including ethnicity, disability, communication needs, type of housing, owner status, use of portable heating, how many people live in the household, and what age they are. We note all those meeting referral criteria, and the proportion that consent to being referred to their local Falls clinic. We are capturing data for all those who attend a subsequent Falls Clinic appointment by monitoring the Falls Clinics lists. We note any reason for non-attendance where it is available. The next stage of the patient's journey through the health service following their initial Falls service appointment is noted from Falls service reports. This includes referral of study patients to other services such as ophthalmology; enrolment in an exercise programme; or immediate discharge from clinic. If the participant is immediately discharged, we will note why immediate discharge occurred, where this information is available, in order to monitor the effectiveness of referral from Home Fire Safety Visits.

#### Objective 3

(i) The number of Falls clinic patients given information about domestic fires and the Home Fire Safety Visit scheme

(ii) Number and percentage of Falls clinic patients over 60 years old (or their advocate) in Southwark and Lambeth who contact LFB's central call centre to arrange a Home Fire Safety Visit.

(iii) Number and percentage of study participants who receive a Home Fire Safety Visit.

Specifically, we are recording the demographic data (age, postcode) of patients who receive a fire safety booklet and freepost Home Fire Safety Visit invitation card, and by whom it was given (nurse or doctor). We use the dedicated study code to monitor how many Visits were booked for older people attending the Falls Clinics, and how many Visits were completed. Additionally, using the subsequent Home Fire Safety Visit assessment data, we are able to monitor whether older people attending Falls Clinics were additionally at risk of accidental domestic fire.

We are collecting the fire and falls assessments for all participants. Using simple analyses of data we will investigate the demographic and socioeconomic characteristics of our study participants and search for associations between older people's risk of fire and falling, and contact with Falls Clinics.

#### Objective 4

In order to examine the acceptability of the intervention introduce during our project, we are conducting short (approximately 30 minute) semi-structured interviews with (i) older service users, (ii) Brigade and (iii) Falls service staff, to assess the perceived ease, value and acceptability of the intervention. Participants are being chosen to represent the diversity of users and stakeholders. These interviews are audio recorded and transcribed verbatim. Data analysis will identify both positive and negative key issues and themes of importance to participants.

### Project advisory group

Stakeholders' and users' ongoing involvement are integral to the successful execution of this proof of concept study. A representative fire fighter from LFB, a hospital Falls clinical lead, and a PCT falls clinical lead (responsible for coordination and clinical governance of the Falls services) have joined us in a project advisory group with a user of the Southwark and Lambeth Falls services, the director of the Social Care Workforce Research Unit, King's College London, and the Social Policy Research Manager in the Policy and External Affairs Department of Age UK. The group are meeting with the research team regularly over the life of the project to provide advice and additional expertise.

### Ethical considerations

This study has been approved by the St Thomas' Hospital (local) Research Ethics Committee (REC reference number 08/H0802/103). All participants are given information leaflets about the study. Those undergoing a falls assessment as part of their booked Home Fire Safety Visit, and/or being interviewed about their views and experiences of the study are also asked to sign a consent form. If any individual appears unable to understand the information about the project following detailed discussions with the researcher, data collection and/or interviewing will be terminated.

## Discussion

The recent Local Government and Public Involvement in Health Act (2007) requires all public sector partners, including Fire and Rescue Authorities in England, to engage with and deliver a shared agenda for their communities. This is currently to be led by the local authority in conjunction with the Local Strategic Partnership, a non-statutory partnership that provides an overarching local co-ordination framework within which other partnerships can operate, although this may be subject to change under the new coalition government. A key output of this Partnership is a Sustainable Community Strategy; a long-term planning document for improving the quality of life and services in a local area.

Local authorities additionally have to prepare a Local Area Agreement (LAA) based on their Sustainable Community Strategy. The LAA will then be used to deliver an agreed set of priorities based on central government's national priority outcomes for local government across the range of its services. To support the delivery of these national and local priorities, the previous government established a range of approximately 180 national indicators [37] covering a range of local services including health and fire prevention.

The testing of our model of partnership working between Fire and Rescue Services and health services in England is especially relevant considering the urgent need to reduce public service costs while improving outcomes, a significant and on-going challenge for public sector organisations. Reductions in public sector funding means we need to devise new ways of working to achieve our aims whilst protecting or improving efficiency and effectiveness. This may be achieved through partnership working between those who have historically not worked together, or between those who currently work together in different contexts. For example, joint working between the Fire and Rescue Services and health services in the UK has traditionally focused on responding to community-based emergencies rather than on prevention strategies. However, in many parts of the country this is now changing, with more Fire and Rescue Services working with partners in their local community in a preventative capacity to ensure the early identification, safety and wellbeing of the most vulnerable populations.

We believe our study is the first intervention that aims to investigate whether two public sector services jointly working to reduce the risk of falling and accidental domestic fire is of mutual benefit to both public service staff and local communities. It fits absolutely the requirement to deliver a shared agenda for the communities serviced by both LFB and specialist NHS services and national policy such as the National Service Framework for Older People [14].

We anticipate that this proof of concept study will demonstrate that joint working between fire service and health service staff can be effective and acceptable to users and stakeholders, and can improve the safety and wellbeing of vulnerable older people by early identification of those previously unknown to services. If this is the case, we envisage advocating this model of working more broadly to fire services and health services throughout the UK. Part of our discussions with key stakeholders will be to ascertain how this joint working can be taken forward after the proof of concept study has ended. For example, would it be acceptable for fire fighters to become trained in administering a short falls assessment and make judgements about clinical referral; would it be acceptable for community therapy teams to routinely liaise with fire services to identify older residents; or is there a third way to roll out a joint working initiative that meets local and national government objectives?

## Competing interests

The authors declare that they have no competing interests.

## Authors' contributions

KL conceived and designed the study protocol, led the funding and Ethics Committee applications and prepared the first draft of the manuscript. AHL participated in the development of the study protocol design and in gaining ethics committee approval. DGW participated in the conception of the study and its design and coordination. FCM participated in the design of the study and its coordination. All authors read and approved the final manuscript.

## Pre-publication history

The pre-publication history for this paper can be accessed here:

http://www.biomedcentral.com/1472-6963/10/327/prepub
